# Data-driven prediction of adverse drug reactions induced by drug-drug interactions

**DOI:** 10.1186/s40360-017-0153-6

**Published:** 2017-06-08

**Authors:** Ruifeng Liu, Mohamed Diwan M. AbdulHameed, Kamal Kumar, Xueping Yu, Anders Wallqvist, Jaques Reifman

**Affiliations:** Department of Defense Biotechnology High Performance Computing Software Applications Institute, Telemedicine and Advanced Technology Research Center, U.S. Army Medical Research and Materiel Command, Fort Detrick, MD 21702 USA

**Keywords:** Drug-drug interactions, Adverse drug reactions, Drug-protein interactions, Synergistic drug-drug interactions, Pharmacovigilance

## Abstract

**Background:**

The expanded use of multiple drugs has increased the occurrence of adverse drug reactions (ADRs) induced by drug-drug interactions (DDIs). However, such reactions are typically not observed in clinical drug-development studies because most of them focus on single-drug therapies. ADR reporting systems collect information on adverse health effects caused by both single drugs and DDIs. A major challenge is to unambiguously identify the effects caused by DDIs and to attribute them to specific drug interactions. A computational method that provides prospective predictions of potential DDI-induced ADRs will help to identify and mitigate these adverse health effects.

**Method:**

We hypothesize that drug-protein interactions can be used as independent variables in predicting ADRs. We constructed drug pair-protein interaction profiles for ~800 drugs using drug-protein interaction information in the public domain. We then constructed statistical models to score drug pairs for their potential to induce ADRs based on drug pair-protein interaction profiles.

**Results:**

We used extensive clinical database information to construct categorical prediction models for drug pairs that are likely to induce ADRs via synergistic DDIs and showed that model performance deteriorated only slightly, with a moderate amount of false positives and false negatives in the training samples, as evaluated by our cross-validation analysis. The cross validation calculations showed an average prediction accuracy of 89% across 1,096 ADR models that captured the deleterious effects of synergistic DDIs. Because the models rely on drug-protein interactions, we made predictions for pairwise combinations of 764 drugs that are currently on the market and for which drug-protein interaction information is available. These predictions are publicly accessible at http://avoid-db.bhsai.org. We used the predictive models to analyze broader aspects of DDI-induced ADRs, showing that ~10% of all combinations have the potential to induce ADRs via DDIs. This allowed us to identify potential DDI-induced ADRs not yet clinically reported. The ability of the models to quantify adverse effects between drug classes also suggests that we may be able to select drug combinations that minimize the risk of ADRs.

**Conclusion:**

Almost all information on DDI-induced ADRs is generated after drug approval. This situation poses significant health risks for vulnerable patient populations with comorbidities. To help mitigate the risks, we developed a robust probabilistic approach to prospectively predict DDI-induced ADRs. Based on this approach, we developed prediction models for 1,096 ADRs and used them to predict the propensity of all pairwise combinations of nearly 800 drugs to be associated with these ADRs via DDIs. We made the predictions publicly available via internet access.

**Electronic supplementary material:**

The online version of this article (doi:10.1186/s40360-017-0153-6) contains supplementary material, which is available to authorized users.

## Background

Adverse drug reactions (ADRs) caused by individual drugs and drug combinations constitute one of the top 10 causes of death in the United States [1]. To alleviate this problem, ADRs caused by a single candidate drug are closely monitored and investigated during the drug development and approval process [2]. For instance, drug candidates are routinely tested in many in vitro and in vivo assays to predict their toxicity to humans [3]. Similarly, subsequent clinical trials usually focus on a single drug and its therapeutic effect against a particular disease or condition. Phase I clinical trials investigate the safety profile of a candidate drug on a small group of volunteers. Phase II trials evaluate its safety and efficacy in a larger group of volunteers. Finally, in Phase III clinical trials, these effects and ADRs are monitored in a large group of selected volunteers. Drugs that successfully pass these hurdles can then be approved for general clinical use.

In real-life settings, however, many patients, especially the elderly, are on multiple prescriptions and over-the-counter medications for treating different and usually unrelated conditions. ADRs due to interactions of co-administered drugs constitute more than 20% of all reported ADRs [4] and are clinically relevant in up to 80% of elderly cancer patients [5]. These effects are rarely discovered or systematically investigated during drug development because of the extensive scope of the problem. In fact, although most ADRs induced by individual drugs have been discovered and carefully monitored in clinical trials before drug approval, information on nearly all ADRs induced by drug-drug interactions (DDIs) has been generated after drug approval [6]. This poses continuous and serious risks to patients’ health.

Co-administration of two drugs can have multiple outcomes concerning adverse drug effects. The adverse effects associated with either drug could be enhanced, or a new unanticipated effect not previously associated with either drug could arise. They represent synergistic DDIs. Alternatively, a positive outcome may occur when a drug counteracts the adverse effect of the other, although this is not captured in ADR reporting systems.

Several methods to computationally predict DDIs have recently been published. Vilar et al. developed a method based on the structural similarity of drug molecules [7]. They hypothesized that if *drug A* interacts with *drug B*, and *drug C* is structurally similar to *drug A*, then *drug C* is also likely to interact with *drug B*. Gottlieb et al. developed a more elaborate similarity-based approach for inferring DDIs [8]. In addition to the structural similarity between drugs, they defined several other similarities, including drug–target similarity, drug–side effect similarity, drug–target protein sequence similarity, as well as semantic similarity measures based on either gene ontology or the Anatomical Therapeutic Chemical (ATC) classification. They combined the similarity measures to generate 49 features for each drug pair, and then used the features to train a logistic regression classification model for predicting DDIs. Huang et al. published an algorithm for predicting pharmacodynamic DDIs based on the closeness of drug targets in a human protein-protein interaction network [9]. Their hypothesis is that drug pairs with a minimum distance of 0 in a protein-protein interaction network are those sharing at least one overlapping target and, therefore, have a high probability of DDIs. Recently, Cami et al. constructed a DDI network for predicting unknown DDIs [10]. They collected DDI information from Multum VantageRx™, a drug safety database, and constructed a DDI network by representing each drug as a node and each DDI as an edge connecting two drugs. They then defined covariates of DDIs from the DDI network architecture, ATC taxonomy, and molecular substructures of drugs, and performed logistic regression analyses by using these covariates as variables to predict novel DDIs. More recently, Luo et al. published a method for predicting DDIs based on docking simulations of drugs into well-defined binding sites of 611 human proteins [11]. After we submitted this paper, Noor et al. published a paper on a novel pharmacovigilance inferential framework to infer mechanistic explanations for asserted drug-drug interactions (DDIs) and to deduce potential DDIs [12].

Although all of the published methods predict the likelihood of DDIs between two drugs, none explicitly predicts the resulting ADR effects. Furthermore, they do not distinguish synergistic from antagonistic DDI effects, i.e., those that reduce the severity of single-drug ADRs. From a practical viewpoint, however, the ability to predict both the possibility of DDIs and their adverse health effects is crucially important.

We recently developed a computational approach for predicting the therapeutic potential of marketed drugs based on genome-wide drug-protein interaction profiles called the drug-protein interaction profile-based repurposing (DPIR) method [13]. Because both the desirable therapeutic and undesirable adverse effects are drug-induced effects in humans, we can also apply this method to predict ADRs caused by individual drugs. In the present study, we expanded this method to predict potential DDI-induced ADRs and carried out detailed cross-validation calculations to demonstrate the performance and reliability of the method. The results indicated that the approach was robust with respect to false positives and false negatives in the training set. We therefore made predictions of DDI-induced ADRs for all pairwise combinations of 764 commercially available drugs. The results are publicly available as a searchable database at http://avoid-db.bhsai.org.

We also highlighted the utility of the ADR models developed in this study by examining the overall aspects of DDI-induced ADRs across 764 drugs and identifying ADRs among combinations of specific therapeutic drug classes. The predictions can be used to examine drug alternatives that avoid ADRs induced by specific combinations of two classes of drugs (e.g., between an anti-infective drug and an anti-convulsant drug). We further used the models to prospectively examine drug combinations that could induce bladder cancer and identified combinations of pioglitazone and statins as potential causes of concern [14].

## Methods

### Assumptions and definition of synergistic ADRs

Our approach is based on the assumption that drug-protein interactions can be used as independent variables in predicting ADRs. Although not all drugs exert their therapeutic effect by directly binding to or modulating protein targets, all drugs, once administered, systemically interact with an array of human proteins. These interactions can affect protein function either directly (inhibition, activation, allosteric regulation, etc.) or indirectly by modulating one or more proteins in pathways that affect physiological processes (signaling, repair, apoptosis, etc.) that could ultimately be manifested as a systemic reaction (nausea, dizziness, heart disease, etc.). The mechanisms of these interactions are largely unknown, and their complexity defies a simple functional description based on drug-protein interactions.

**Fig. 1:**
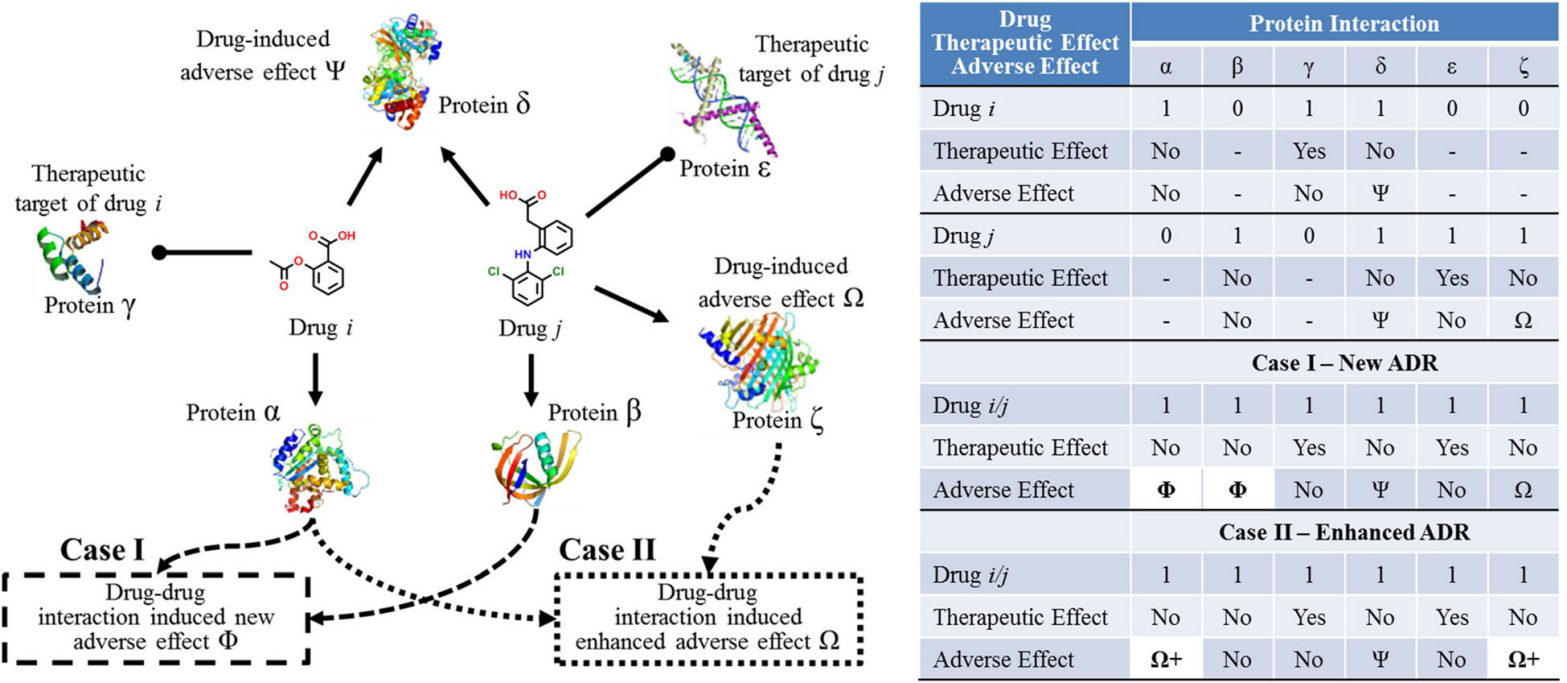
Schematic illustration of the drug-protein interactions necessary for drug-drug interaction (DDI)-induced adverse drug reactions (ADRs). Drugs *i* and *j* interact with proteins α, β, γ, δ, ε, and ζ to induce both therapeutic effects as well as adverse effects Ψ, Ω, and Φ. In Case I, simultaneous drug interaction with both proteins α and β is necessary for a DDI to induce ADR Φ. Because drug *i* interacts with α but not β and drug *j* interacts with β but not α, no DDI occurs when the two drugs are administered individually. However, when the two drugs are co-administered, the requirement of simultaneous drug interaction with both α and β, and hence the condition for DDI-induced ADR Φ, are satisfied. In Case II, an existing adverse effect Ω caused by drug *j* is enhanced by drug *i* interacting with α, aggravating the adverse effect to a degree that is not possible by drug *j* alone

We define a synergistic DDI-induced ADR as arising from the simultaneous interactions between co-administered drugs and their protein targets, which cause a new or enhanced ADR beyond what either drug can trigger on its own. Figure 1 schematically illustrates this concept with two drugs that individually interact with a number of proteins, each causing both therapeutic effects and adverse reactions when administered alone. When the two drugs are given together, more than one scenario is possible. In particular, we were interested in cases where the adverse reaction is triggered or enhanced by multiple protein targets activated by two different drugs. We illustrate these cases in Fig. 1, where Case I represents the induction of a new adverse effect Φ and Case II the enhancement of a previously known adverse effect Ω. Note that in the case of two drugs having the same interaction profile (drugs *i* and *j* targeting protein δ to cause the adverse effect Ψ in Fig. 1), a synergistic ADR cannot occur.

In Case I, α and β represent two proteins required to generate the DDI-induced ADR Φ, with drug *i* interacting with α but not β and drug *j* interacting with β but not α. Thus, taking each drug individually does not activate the effect Φ. However, taking drugs *i* and *j* together leads to simultaneous and separate drug interactions with each protein, and hence the induction of Φ.

In Case II, ζ represents a protein that can induce the adverse reaction Ω through its interaction with drug *j*. Co-administering drug *i*, which interacts with α, leads to an enhancement of the original ADR Ω beyond what is achieved by drug *j* alone. Thus, taking the drugs together synergistically enhances the effect Ω.

The key methodological steps we devised to create predictive models of DDI-induced ADRs included *1*) creating a scoring scheme that describes adverse reactions, based on known drug-protein interactions; *2*) defining an appropriate model to capture synergistic interactions; *3*) creating drug-protein interaction profiles for marketed drugs; *4*) defining the protein interaction profiles for a drug combination; and *5*) parameterizing the models by using existing clinical data. Furthermore, we developed a searchable Web-accessible database of predicted DDI-induced ADRs of all commercially available drugs for which drug-protein interaction information is available.

### Scoring drug-protein interactions for an adverse health effect

To predict drug-induced effects, the DPIR method exploits large-scale drug-protein interaction information in the public domain. To estimate the likelihood that a drug-protein interaction contributes to a therapeutic or a side effect, we implemented the Laplacian-corrected estimator used by Xia et al. [15]. Suppose there are N drugs, M of which induce a specific ADR Φ. The baseline probability of a random drug inducing Φ is then M/N. Let us further assume that N_B_(*α*) of N drugs interact with a specific protein *α*, and N_A_(*α*) of N_B_(*α*) drugs induce the effect Φ. The probability that these drug-protein interactions are responsible for the effect Φ is then estimated as N_A_(*α*)/N_B_(*α*). This is not a statistically reliable estimate when few drugs interact with protein *α*. For instance, when N_B_(*α*) = 1 and N_A_(*α*) = 1, the probability is 100%, which is clearly an overestimate due to under-sampling of this drug-protein interaction. When more drugs interacting with this protein are included, the probability estimate becomes more realistic. To correct for under-sampling, we considered adding K virtual drugs that interact with this protein. A reasonable estimate of the number of virtual drugs that induce Φ is K × M/N, which adjusts the estimated probability of the drug-protein interactions responsible for the effect Φ to (N_A_(*α*) + K × M/N)/(N_B_(*α*) + K). This modification ensures that when the drug-protein interaction is extremely under-sampled, i.e., N_B_(*α*) → 0, the probability estimate approaches the baseline probability, M/N. In our study, we used K *=* N/M so that the probability estimate was expressed as (N_A_(*α*) + 1)/(N_B_(*α*) + N/M). When this probability estimate is higher than the baseline probability, M/N, the drug-protein interaction enhances the ADR. Conversely, when it is lower, the drug-protein interaction reduces the ADR.

On the basis of this estimate, we defined the protein weight

1 $$ {\mathrm{w}}_{\alpha}= \log \left[\left({\mathrm{N}}_{\mathrm{A}}\left(\alpha \right)+1\right)/\left({\mathrm{N}}_{\mathrm{B}}\left(\alpha \right)+\mathrm{N}/\mathrm{M}\right)\right]\hbox{--} \log \left[\mathrm{M}/\mathrm{N}\right], $$

as the contribution of a drug interaction with protein *α* to the drug-induced effect. Once the weights of all proteins are determined by using a training data set for a particular ADR Φ, we can assess the likelihood that another drug induces the same ADR Φ*.* If *g*_*α*_(*d*_*i*_) represents the interaction of drug *i* with protein *α* and assumes a value of 1 or 0 depending on the presence or absence, respectively, of an interaction, then each interaction contributes *s*_*α*_ = *g*_*α*_(*d*_*i*_) *× w*_*α*_ to the effect, and the total score *S*(Φ, *d*_*i*_) is constructed by summing the contributions of each protein. The higher this score for a drug, the more likely it is to induce the same ADR Φ.

Compared to machine learning algorithms, the scoring model described above has an important advantage in that the only parameters to be determined are the weights of the drug-protein interactions. Unlike most other methods that determine model parameters by minimizing an error function, we determine the model parameters by a simple process. The process only requires *1*) counting the total number of drugs, N; the number of positive drugs, M; the number of all drugs interacting with protein *α*, N_B_(*α*); and the number of positive drugs interacting with protein *α*, N_A_(*α*); and *2*) calculating the weight of protein α, w_*α*_, according to equation (1). Because no parameter minimization is involved, this process is computationally efficient and the number of proteins that can be used is unlimited.

### Scoring synergistic DDIs for an adverse health effect

The same approach can also be used to estimate the likelihood of a pair of co-administered drugs *i* and *j* causing an ADR by replacing the drug-protein interaction with the joint interaction of protein *α* with the co-administered drugs *g*_*α*_(*d*_*i*_) *→ g*_*α*_(*d*_*i*_ *d*_*j*_), *g*_*β*_(*d*_*i*_) *→ g*_*β*_(*d*_*i*_ *d*_*j*_), etc. We denoted the profile of these interactions across all proteins as ***ĝ***(*d*_*i*_) or ***ĝ***(*d*_*i*_ *d*_*j*_). Because antagonistic DDIs reduce the severity of ADRs, their effects are less likely to be reported and collected in ADR reporting systems. Therefore, DDI-induced ADR data should consist mostly of data on synergistic DDI-induced ADRs. By definition, the severity level of a synergistic DDI-induced ADR should be higher than the sum of the ADR severity levels induced by individual drugs. However, there is no straightforward definition of the sum of ADR severity levels, and the relationship between the pharmacology of drug action and the severity of a drug-induced effect (represented by the reporting ratio) is non-linear. Consequently, we require the score of a synergistic DDI-induced ADR to be at least higher than the highest score of the individual drugs, i.e.,

2 $$ S\left(\varPhi, {d}_i{d}_j\right)> \max \left[ S\left(\varPhi, {d}_i\right), S\left(\varPhi, {d}_j\right)\right]. $$

We therefore defined the synergistic DDI score as follows:

3 $$ D D I\left(\varPhi, {d}_i{d}_j\right)= S\left(\varPhi, {d}_i{d}_j\right)\hbox{--} \max \left[ S\left(\varPhi, {d}_i\right), S\left(\varPhi, {d}_j\right)\right], $$

where *DDI*(Φ,*d*_*i*_ *d*_*j*_) must be higher than 0 for a synergistic DDI-induced ADR. The higher the DDI score, the more severe is its effect.

### Data for drug-protein interaction profile *ĝ*(*d*_*i*_)

To create large-scale drug-protein interaction profiles, we downloaded all protein-chemical links from the STITCH database [16]. This database (STITCH 4.0) contains chemical-protein interaction information, derived from a broad range of sources, between 300,000 small molecules and 2.6 million proteins from 1,133 organisms. Each entry of the database is associated with a confidence measure, calculated by $1-{\prod}_n\left(1-{c}_n\right)$, where ${c}_n$ denotes the confidence of the interaction from the *n*th information source. In the STITCH database, scores of 0.40–0.70, 0.70–0.90, and 0.90–1.00 indicate medium, high, and highest confidence levels, respectively.

To remove low-confidence chemical-protein interactions, we filtered out entries in STITCH 4.0 with confidence scores lower than 0.40. In addition, we only retained entries of chemical interactions with human (*Homo sapiens*) proteins. These two steps reduced the total number of chemical-protein interaction entries from >171 million to just over a half million. The categories of chemical-protein interactions with the highest occurrence in the database are binding (chemical binds to protein), inhibition (chemical inhibits protein function), activation (chemical enhances protein function), and catalysis (chemical is a substrate of metabolic proteins). To create drug-protein interaction profiles relevant for predicting drug-induced effects in humans, we only retained interactions from these four categories.

We used a binary string of 0 and 1 s to encode the chemical-human protein interaction information of a drug contained in the STITCH database. Each protein has four designated positions in the string, with a 1 or 0 at each of the positions representing whether or not the drug binds to the protein, activates the protein, inhibits the protein, or is metabolized by the protein, respectively.

### Definition of drug pair-protein interaction profile *ĝ*(*d*_*i*_*d*_*j*_)

**Fig. 2:**
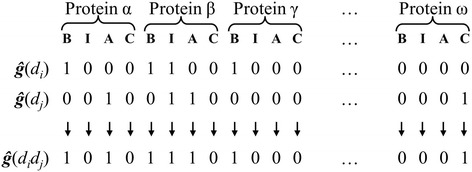
Binary bit string representation of genome-wide drug-protein interaction profiles of individual drugs and drug combinations. In the bit strings, the drug interaction with each protein is encoded by four bits representing binding (*B*), inhibition (*I*), activation (*A*), and catalysis (*C*). For any drug, if information for an interaction is present in the STITCH database with at least a medium confidence level, the corresponding bit in the string is turned on (assigned a value of 1); otherwise, it is turned off (assigned a value of 0). To generate a drug pair-protein interaction profile ***ĝ***(*d*_*i*_ *d*_*j*_) from the constituent drug-protein interaction profiles ***ĝ***(*d*_*i*_) and ***ĝ***(*d*_*j*_), we implemented the logical OR operation. Thus, we turned the bit off when neither drug interacts with a protein; otherwise, we turned it on when interactions for either or both drugs are present

Because each drug targets different proteins for different therapeutic effects and is likely to be associated with any number of off-targets, taking multiple drugs simultaneously is likely to affect more proteins than taking a single drug alone. Thus, a pair of drugs *i* and *j* should have a protein interaction profile ***ĝ***(*d*_*i*_ *d*_*j*_) more densely populated by 1 s than the corresponding individual drug-protein interaction profiles ***ĝ***(*d*_*i*_) and ***ĝ***(*d*_*j*_). We created drug pair-protein interaction profiles by applying the logical OR operator on the constituent drug-protein interaction profiles, as schematically illustrated in Fig. 2. This approach reduces the multiple types of interaction in each classification category to a single “interaction” that does not take into account additional complexities (e.g., site-competition, or non-additive effects at the molecular level). In essence, the total number of proteins a drug pair interacts with is the sum of the unique protein interactions from both drugs.

### Data for ADRs induced by synergistic DDIs

To evaluate the performance of our approach, we used the TWOSIDES database of Tatonetti et al. as the information source for ADRs induced by synergistic DDIs [17]. This database contains 868,221 significant associations between 59,220 pairs of drugs and 1,301 adverse events that cannot be clearly attributed to either drug alone, as well as 3,782,910 significant associations for which the drug pairs have higher side-effect association scores than those of the individual drugs alone. These adverse events correspond to the definition of ADRs induced by synergistic DDIs, for which we were interested in developing predictive models. These data indicate that about 23% (868,221/3,782,910) of the DDI-associated ADRs may be considered novel, because they were reported only when a drug pair was taken and not when either drug was taken alone.

The TWOSIDES drug pair-ADR associations we used to build the prediction models were derived from statistical analyses of pharmacovigilance signals. As such, they should not be considered as causal associations because most of them may not have been validated in clinical studies. Although the extensive pre-clinical and clinical drug-development efforts expend considerable resources to minimize toxicity and adverse drug reactions, the DDI-induced adverse effects in the patient population once a drug reaches the market are often initially detected by pharmacovigilance and are largely of idiopathic origin. Naturally, these associations do not imply causation, and may contain false positives. For a method developed to predict associations based on reported data to be useful, it must be robust with respect to the inclusion of false information. As we demonstrate in the next section, detailed cross-validation analyses indicate that moderate amounts of false information in the training data have only a small impact on model performance.

From the drugs in the TWOSIDES database, we identified 645 compounds with unique STITCH compound IDs. However, only 477 of these had drug-protein associations ***ĝ***(*d*_*i*_) with confidence scores of 0.40 or higher. Using these drug-protein interaction profiles, we created drug pair-protein interaction profiles ***ĝ***(*d*_*i*_ *d*_*j*_) for all unique pairwise drug combinations. The profiles contained 2,637 unique human proteins with an initial string length of 10,548 to encode binding, activation, inhibition, and catalysis information. Finally, we shortened the strings by deleting protein-interaction columns only populated by 0 s, resulting in a final string length of 4,135.

### Web-based searchable database of predicted DDI-induced ADRs

We created a Web-based AdVerse effects Of Interacting Drugs Database (AVOID-DB) to allow for interactive query of our predicted DDI-induced ADRs. We developed the AVOID-DB and Web-based graphical user interface (GUI) by using a three-tier software architecture comprising a backend database, controller, and presentation tiers. An Oracle database server using a relational schema stores over 35 million DDI-induced ADRs for fast searching. The controller and presentation tiers implement the search logic and GUI, respectively. We developed the controller and presentation tiers by using Java Platform, Enterprise Edition 7, JavaServer Faces 2.2, and PrimeFaces 6.0 technologies. We designed the Web-based GUI to be responsive to both desktop and mobile device Web-browsers. The GUI uses standards supported by modern Web browsers.

## Results and Discussion

As described above (see Methods), we created drug and drug-pair protein interaction profiles by using data from the STITCH database [16], determined the contribution of each protein to a DDI-induced ADR by using data in the TWOSIDES database [17], and created models for synergistic DDI-induced ADRs. We first evaluated the models constructed and their performance with respect to false positives and false negatives in the training data, and determined the conditions for implementing robust and predictive models for commercially available drugs. Next, we applied the models to examine the ADRs among the drugs and between specific drug classes, as well as drug combinations that cause specific ADRs.

### Assessment of robustness

To evaluate our method, we trained DDI-induced ADR models by using different input sets to examine the impact of varying the training set composition. This is important because DDI-induced ADR information is noisy, given that it is generated from patient and physician reports instead of being derived from carefully designed clinical trials. False positives are unavoidable and true negatives of high confidence are rare. A report of an ADR induced by DDIs between two drugs indicates that the drugs were co-administered and likely induced the ADR via a DDI. In contrast, the absence of a report of an ADR attributed to a drug pair may mean that no DDI exists for the ADR (a true negative); the ADR requires some time to develop or be recognized under co-administration conditions (a false negative); or the two drugs have yet to be co-administered (an unknown). Thus, strictly speaking, there are few if any true negative samples for training and evaluating the models. To overcome this issue, some studies have developed and evaluated DDI prediction models by using reported DDI-inducing drug pairs as positives and an equal number of randomly made-up drug pairs as negatives [8, 9, 11]. In this study, we trained each ADR model by using drug pairs positive for the ADR as positive samples and all other drug pairs in the TWOSIDES database as putative negatives. We designated the putative negatives as “baseline samples” to recognize that they may contain heretofore unknown positives. Thus, the resulting models were aimed at discriminating positive samples from the background baseline samples. The premise underlying this approach is that the number of drug pairs positive for an ADR is always a small fraction of the number of all drug pairs. Thus, even if the baseline class contains unrecognized drug pairs that are also positive for the ADR, the number of positive drug pairs is negligibly small compared with the total number of baseline samples. To assess the robustness of this approach, we varied the number of true positive samples in the training set, as well as the amounts of false negatives and putative false positives in the training set, to investigate the impacts of these variables on model performance.

**Fig. 3:**
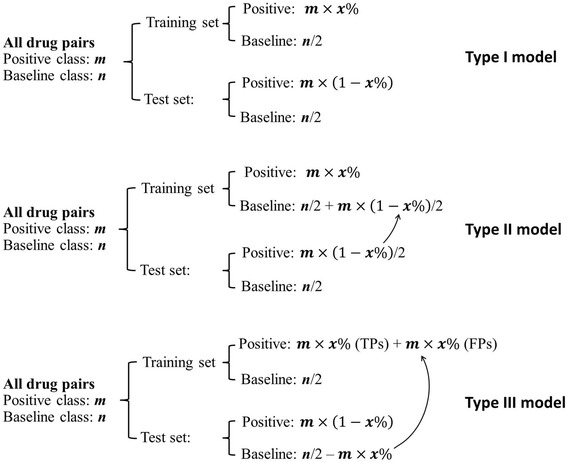
Sample selection for training and evaluation of three types of model (I–III). For type I models, we used 10–90% of all drug pairs positive for an ADR as the positive class for model training, with half of the other drug pairs in the TWOSIDES database used as the baseline class. We used the remaining drug pairs as a test set for assessing model performance. Sample selection for type II models was the same as that for type I models except that we moved half of the positive samples in the test set into the baseline class of the training set to provide known false negatives. Sample selection for type III models was the same as that for type I models except that some randomly selected baseline samples in the test set were moved into the positive class in the training set to provide false positives. The number of false positives was equal to the number of true positives in the training set

Figure 3 shows our experimental design of using different training and test set samples to assess the effects of including inadequate or false information in model development. Type I models probe the effect of the number of positive samples in the training set; type II models investigate the impact of false negatives; and type III models examine the impact of putative false positives.

To develop type I models, we randomly selected 10, 30, 50, 70, or 90% of the drug pairs associated with a DDI-induced ADR as positives for inclusion in the training sets, together with half of the baseline samples. For each selection, we used the remaining drug pairs as a test set for calculating the area under the receiver operating characteristic curve (AUC). The AUC values range from 0.5, which corresponds to a random model without any predictive power, to 1.0, which corresponds to a perfect model. Sample selection for type II models was similar to that for type I models, except that half of the test set positives were assigned as baseline samples of the training set for model development, i.e., we artificially enhanced the number of false negatives in the training set. Sample selection for type III models was again similar to that of type I models, except that the training set positives were composed of a percentage of true positives and an equal number of false positives taken from the test set baseline samples, i.e., we artificially enhanced the number of false positives in the training set. To derive statistically reliable results, we repeated the process of building a model and calculating its AUC 50 times for each type of model and training-set-sample composition.

**Fig. 4:**
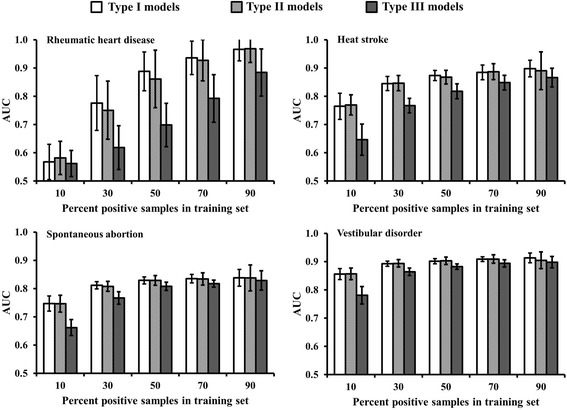
Area under the receiver operating characteristic curve (AUC) derived from cross-validation studies by using three types of model (I–III) for the following adverse drug reactions (ADRs): rheumatic heart disease (Unified Medical Language System code C0035439), heat stroke (C0018843), spontaneous abortion (C0000786), and vestibular disorder (C0042594). These ADRs were correspondingly associated with 78, 331, 897, and 1022 drug pairs. For all panels, the horizontal axis represents the percentage of positive samples for an ADR used in the model training. The error bars show ±1 standard deviation from 50 simulations, using randomly selected training and test set samples

We applied the experimental design described above to four medically significant ADRs selected from the TWOSIDES database, which collectively cover a broad range of positive drug pairs: rheumatic heart disease (Unified Medical Language System (UMLS) code C0035439), 78 drug pairs; heat stroke (C0018843), 331 drug pairs; spontaneous abortion (C0000786), 897 drug pairs; and vestibular disorder (C0042594), 1,022 drug pairs. Figure 4 shows the model performance results, with bar heights representing mean AUC values and error bars indicating ±1 standard deviation. For all four ADRs, AUC values increased with the number of positive samples in the training set, i.e., increasing positive samples in the training set improved model performance. In addition, for all four ADRs, the AUCs of type I models were comparable to those of type II models, indicating that false negatives in the training sets had little impact on model performance. On the other hand, false positives had an appreciable negative impact on model performance, especially for type III models built from a small number of positive samples in the training set. However, for all four ADRs, the AUC values of type III models approached those of the corresponding type I and type II models as the number of positives in the training set increased. Hence, the impact of false information diminished by increasing the number of positive samples in the training set.

**Fig. 5:**
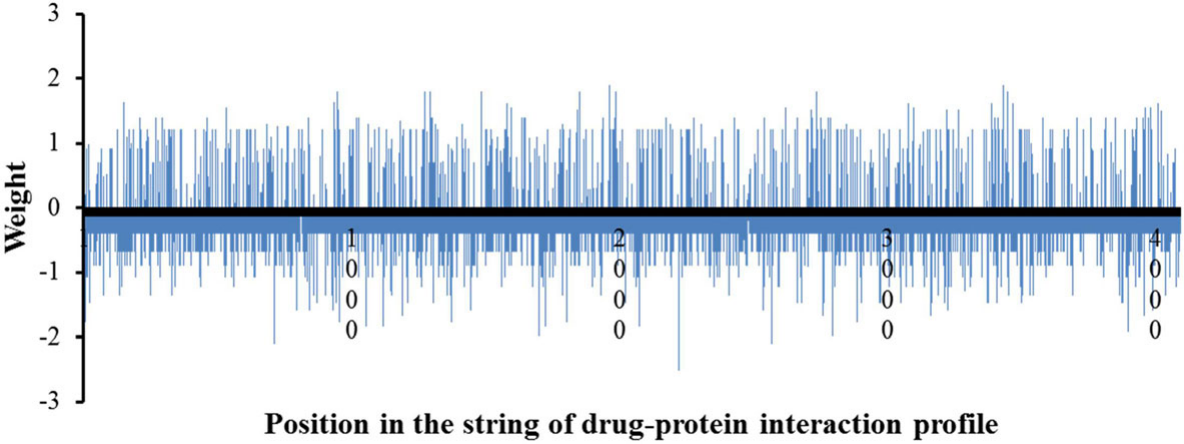
Weights of the drug-protein interactions contributing to the rheumatic heart disease (C0035439) model. The positions on the horizontal axis represent specific drug-protein interactions; the heights of the blue bars denote the weights of the drug-protein interactions in the ADR model. The figure demonstrates that the ADR model is not dominated by only one or a few drug-protein interactions

Given that each drug may interact with a large number of proteins, one concern was that a model may be unduly dependent on one or a very small number of drug-protein interactions. To assess this possibility, we examined the weights of the drug-protein interactions, as defined by equation (1), of the rheumatic heart disease model. The model was built by using the 78 drug pairs as positive training samples and the rest of the drug pairs in TWOSIDES as baseline samples. Figure 5, which shows the numerical weights of all drug-protein interactions of the model, indicates that the model consists of contributions from a large number of drug-protein interactions and is not dominated by one or a few such interactions. In the drug-protein interaction profiles built upon information from the STITCH database with medium and higher confidence drug-protein interactions, a drug interacts with 53 proteins on average, indicating that the drug-protein interaction approach is not biased toward only a few interactions.

### Determination of the minimum number of positive samples required

Insensitivity to false positives and false negatives in the training set is a critical requirement for ADR models, given the uncertain nature of the reported data. In contrast to therapeutic effects that are carefully evaluated in clinical trials, the reporting threshold for drug-induced adverse effects is low. A drug may be associated with an ADR simply because one or more individuals taking the drug experienced the adverse effect, without detailed analyses of potential confounds or other possible causes such as foods or other drugs consumed at the same time. Establishing causal relationships between a drug and ADRs, let alone DDIs to an ADR, is not a trivial matter [17]. Because of the unavoidable presence of false positives and false negatives in the ADR data set, we first needed to determine the minimal information needed to operate our models.

Figure 4 shows that when 90% of all positive samples were used to train the models, the resulting models were highly robust, with AUCs in the 0.8 to 1.0 range. However, when fewer positive samples were used, model performance appreciably declined, especially for the rheumatic heart disease model, which had only 78 positive samples. The AUC of the model built from 10% of the positive samples (8 drug pairs) was between 0.5 and 0.6, close to that of a random model.

Additional cross-validation calculations for a large number of ADRs indicated that the number of positive samples in the training set was the most crucial factor for developing a predictive model. When the training set included less than 50 positive samples, the resulting AUCs of many models fell in the range of 0.5 to 0.6. Thus, we selected the minimum requirement for model building to be at least 50 positive ADR samples.

### Parameterization, evaluation, and instantiation of DDI-induced ADR models

On the basis of the above results, we trained our final models for all ADRs associated with 50 or more drug pairs in the TWOSIDES database and drugs with medium or higher confidence drug-protein interaction information in the STITCH database. To build the final model for an ADR, we parameterized the model using all drug pairs reported to be associated with the ADR in the TWOSIDES database as positives and all other drug pairs in the database as baseline samples. To assess the quality of the resulting models, we performed 10-fold cross-validation for each model. That is, for each ADR, we segregated both positive and baseline drug pairs randomly into 10 groups, and used nine of the groups to train the model and the left-out group to evaluate the resulting model. We repeated this process nine times so that each group was left out once.

**Fig. 6:**
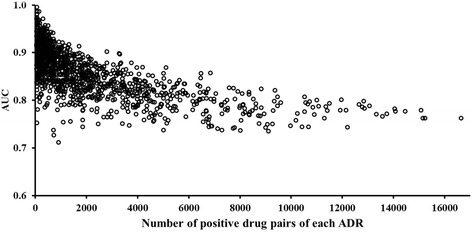
Area under receiver operating characteristic curve (AUC) for 1,096 models of adverse drug reactions (ADRs), estimated from 10-fold cross-validation studies. Each circle represents an ADR prediction model with its AUC value on the vertical axis and the number of drug pairs that induced this ADR on the horizontal axis. The ADR model with the most drug pairs was nausea (Unified Medical Language System code C0027497), with >16,000 constituent pairs

Figure 6 shows that the resulting AUCs fell in the range of 0.7 to 1.0 and generally decreased as the number of drug pairs associated with the ADRs increased. Among the models, 176, 699, and 221 had AUCs in the ranges of 0.7–0.8, 0.8–0.9, and 0.9–1.0, respectively. The average AUC of all models was 0.85 with a standard deviation of 0.05. The general trend of higher AUC values associated with fewer positive drug pairs may be attributed to the ADRs being associated with pharmacologically well-defined drug interactions with a smaller number of specific proteins. Conversely, if an ADR is caused by many drug pairs (e.g., nausea, which is associated with more than 16,000), developing a predictive model with high specificity becomes more difficult because manifestation of the ADR can be traced to a very large number of drug-protein interactions. We also examined whether the reported number of ADRs for a drug pair would affect model performance. The results indicated that model performance, as measured by the AUC, is largely insensitive to the number of reported ADRs, suggesting that the model construction is robust with respect to the propensity of a drug to be associated with ADRs (Additional file 1: Figure S2).

As a measure of model quality, the AUC reflects a model’s ability to enrich positives in high-scoring samples. To make categorical predictions, we needed a DDI score threshold that would classify a drug pair as positive if its score were higher than the threshold and negative otherwise. To be consistent with equation (2), the lowest threshold value is 0. Table 1 shows the AUC, accuracy, sensitivity, and specificity of the four representative ADR models, as well as the average values for all 1,096 prediction models, one for each ADR. The overall categorical predictions showed a mean accuracy of 0.89, a mean sensitivity of 0.63, and a mean specificity of 0.90. These performance measures are adequate for a balanced sample distribution in which the positive and non-positive classes are roughly of equal size. However, the occurrence of DDI-induced ADRs is highly unbalanced. For a specific DDI-induced ADR, only a very small fraction of all possible drug pairs are positive. As a result, a useless model that predicts all drug pairs to be negative for any DDI-induced ADR would have a perfect specificity and very high accuracy. A more practical performance measure for such situations is the positive predictive value (PPV), also known as precision. This is defined as the ratio of the number of true positives (TP) to all predicted positives, including both true and false positives (FP),

**Table 1:** Operational characteristics of adverse drug reaction (ADR) models

Adverse drug reaction	AUC	Accuracy	Sensitivity	Specificity	PPV
Exemplar ADR models					
Rheumatic heart disease	0.97	0.96	0.81	0.96	0.02
Heat stroke	0.90	0.84	0.83	0.85	0.02
Spontaneous abortion	0.84	0.89	0.63	0.89	0.05
Vestibular disorder	0.91	0.91	0.73	0.91	0.07
All 1,096 ADR models					
Mean	0.86	0.89	0.63	0.90	0.09
Standard deviation	0.05	0.05	0.15	0.05	0.08

The performance of models to predict ADRs induced by drug-drug interactions (DDIs) were estimated from 10-fold cross-validation. The values were calculated by using a DDI score threshold of 0.0 for all ADRs. The calculated positive predictive values (PPV) are given for the developed ADR models

4 $$ \mathrm{P}\mathrm{P}\mathrm{V}=\mathrm{T}\mathrm{P}/\left(\mathrm{TP}+\mathrm{FP}\right), $$

and is a measure of the probability that positive predictions truly are associated with the ADR. Table 1 shows that with a DDI score threshold of 0, the PPVs of the four representative ADR models ranged from 2 to 7%, and the average PPV of all 1,096 ADR models was 9%. They appear lower than what might be considered desirable. This is at least partly because the number of positive drug pairs for many ADRs was extremely small compared with the total number of all possible drug pairs. For example, only 78 drug pairs in the TWOSIDES database were associated with rheumatic heart disease. The total number of unique drug pairs was 113,526. Hence, the probability that a randomly selected drug pair will induce this disease is therefore 0.069%. Although the PPV of the rheumatic heart disease model was only 2%, it represented a 29-fold enrichment of positives relative to a uniform distribution of positives among all drug pairs.

**Fig. 7:**
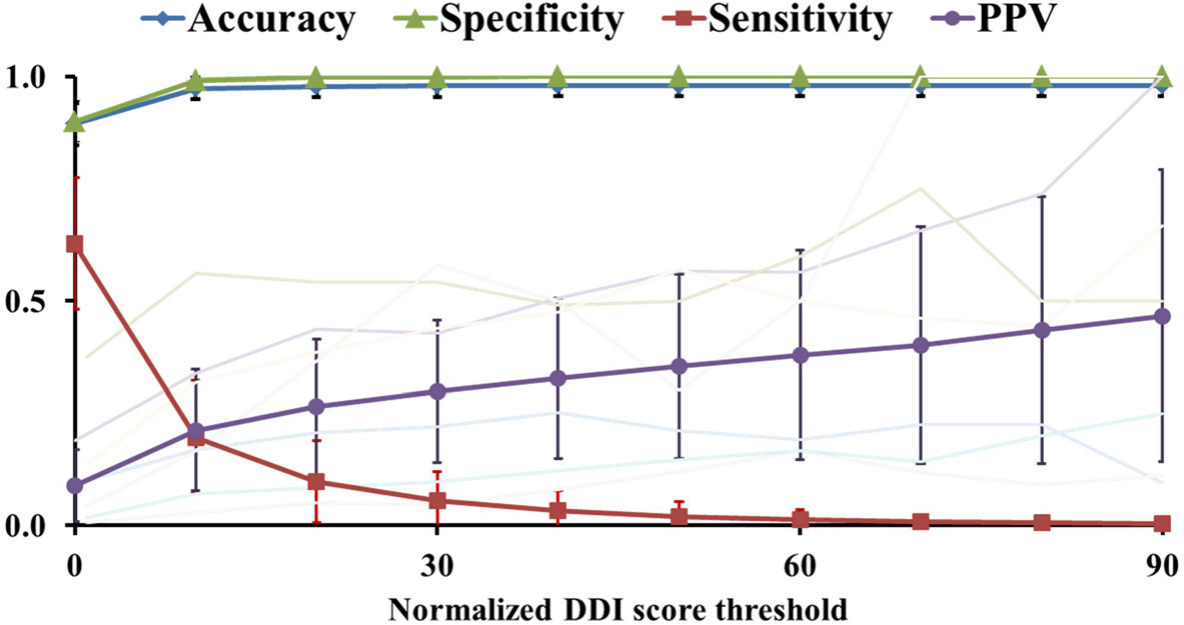
Impact of increasing the normalized DDI score threshold on average measures of prediction performance for 1,096 ADR models evaluated by 10-fold cross validation. The error bars indicate ± 1 standard deviation. The faint lines, which represent PPV traces of several ADRs, show that all initially increase with the DDI score threshold, but some reach a maximum and then fluctuate or drop off with further increases in the DDI score threshold

One parameter that may affect the PPV is the DDI score threshold. To assess its impact, we normalized the DDI scores by dividing each score derived from equation (3) by the maximum score of each model and multiplying it by 100, so that the maximum score of each model was 100. We then calculated all performance measures of each model, using normalized DDI score thresholds of 0, 10, 20, …, and 90 for categorical predictions. Figure 7 shows the average values of model performance measures across all 1,096 ADR models. A threshold of 0 yielded the most balanced performance measures, with the least separation between sensitivity and specificity. For most models, increasing the threshold increased the PPV, as it reduced the number of false positives. However, with an elevated threshold, both the PPV variance and the number of false negatives also increased. As a result, for some models, the PPV initially increased with higher DDI-score thresholds, reached a maximum, and then dropped off with increased threshold values.

### Predictions of DDI-induced ADRs for drugs on the market

The TWOSIDES database contains synergistic DDI-induced ADR information for 645 drugs and 1,318 ADRs. The number of unique pairwise combinations of 645 drugs is 207,690. The possible number of unique drug pair–ADR associations between 207,690 drug pairs and 1,318 ADRs is 274 million, whereas the TWOSIDES database “only” contains 4.7 million drug pair–ADR associations. Thus, this latter information may only represent a small fraction of all possible DDI-induced ADRs. Considering that the total number of United States Food and Drug Administration–approved drugs is well over 1,000, the possibility remains that a large number of DDI-induced ADRs are still unrecognized.

By using our ADR models, we can make predictions for any drug combination, provided that drug-protein interaction information is available, to identify candidate DDI-induced ADRs. We examined 1,332 drugs on the market and used the information in STITCH 4.0 to create drug-protein interaction profiles for 764 drugs. For each pairwise drug combination, we calculated DDI-induced ADR scores by all of the models and provided categorical predictions for whether the drug pairs contribute to ADRs via synergistic DDIs. We thus generated over 36 million positive drug pair–ADR associations. This roughly corresponds to 10% of all possible drug pair–ADR associations. We also used the TWOSIDES data to determine DDI-score thresholds for 10, 20, 30, 40, and 50% PPV predictions via 10-fold cross validation. We then used these DDI-score thresholds to make categorical predictions for pairwise combinations of the 764 drugs so that the predictions could be associated with different PPV-confidence levels.

**Fig. 8:**
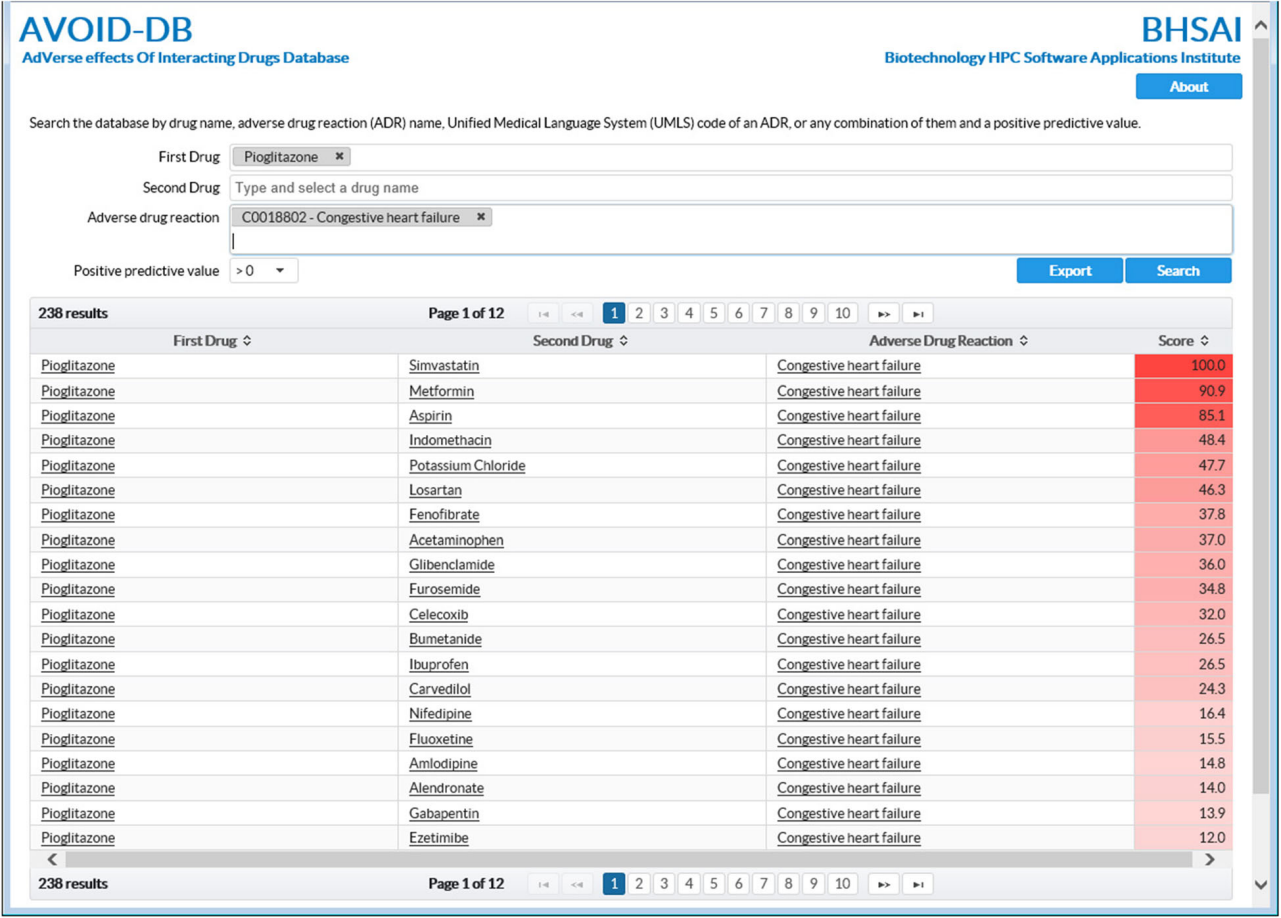
Screenshot of the AdVerse effects Of Interacting Drugs Database (AVOID-DB). The database can be queried with the name of a single drug to retrieve all predicted DDI-induced ADRs associated with the drug, or with two drug names to retrieve all DDI-induced ADRs associated with the two drugs. Similarly, the database can be queried with one or more specific ADRs to retrieve all drug pairs causing the ADRs via synergistic DDIs. The drug and ADR names, which constitute a controlled vocabulary, can be selected from among the available names in the database. When available, the drug and ADR names are cross-linked to DrugBank and National Library of Medicine resources for further information. The normalized *DDI* (Φ,*d*_*i*_ *d*_*j*_)-scores are color-coded from dark to light red for visual guidance. The Web page is accessible at http://avoid-db.bhsai.org

To make our predictions readily accessible, we developed a Web-based query utility for searching the predicted positives for any of the 1,096 ADRs, using one or two of the 764 drug names. Figure 8 shows a screenshot of the application. The data can be queried by using one or two drug names (common synonyms are recognized), the ADR names, the UMLS codes of ADRs, or a combination of drug names and ADRs, as well as the PPV threshold. When the query consists of a single drug, the search returns all combinations of that drug with each of the other 763 drugs, for which the DDI score is positive, from among the 1,096 ADR models. When the query consists of two drugs, the search returns all ADR models that yield positive DDI scores for the drug combination. When the query is based on an ADR name or UMLS code only, the search returns all pairwise drug combinations with positive DDI scores. When available, the results page provides information links to DrugBank [18] for drug information and the National Library of Medicine MedGen resources [19] for adverse health effects. The Web-accessible database is publically available at http://avoid-db.bhsai.org.

### Applications of DDI-induced ADR models

We used the developed models to examine both broader aspects of the occurrence of DDI-induced ADRs among the 764 drugs for which we could make predictions and the susceptibility of particular drug-classes to be generally involved in ADRs.

#### General occurrence of predicted DDI-induced ADRs

**Fig. 9:**
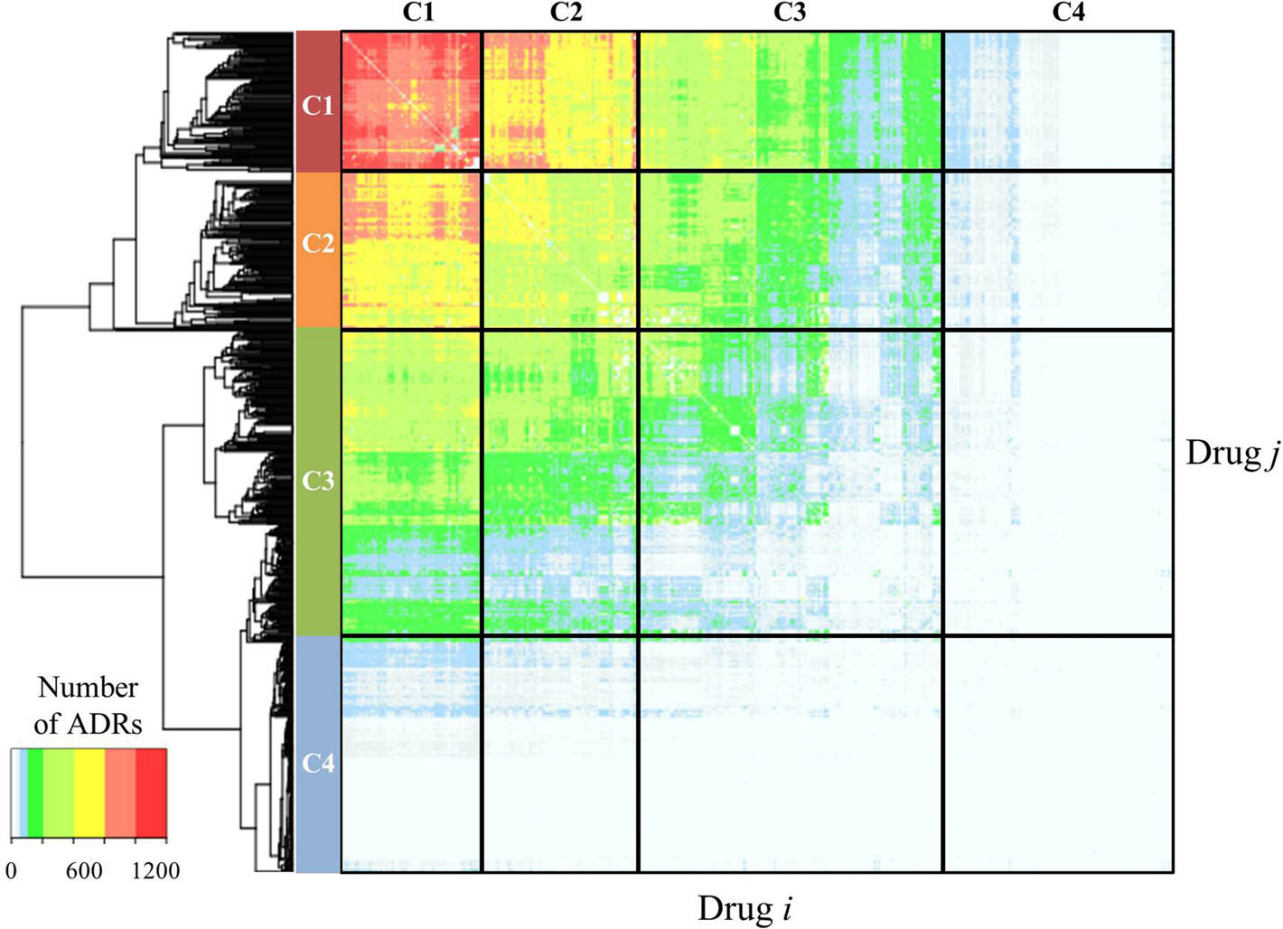
Two-way clustering of predicted DDI-induced ADRs. We included all drugs on the market for which we could make predictions. Each element in the matrix represents the number of ADRs caused by each drug pair. The drugs roughly formed four clusters (C1–C4), where cluster C1 was associated with the most ADRs and cluster C4 with the least

Figure 9 shows two-way clustering of the number of ADRs associated with each possible drug-pair predicted by the ADR models. The data segregated into four groups of drugs (clusters C1–C4). This highlights the observation that certain drugs and drug combinations were associated with different rates of ADR occurrence, indicating inherent differences in their propensity to trigger DDI-induced ADRs. The drugs in clusters C1 and C2 were associated with numerous ADRs (typically greater than ~800 and ~500, respectively), whereas clusters C3 and C4 were, respectively, associated with moderate and small numbers (typically less than ~50 for the latter) of ADRs. The rate of ADR occurrence among drugs can be regarded as a proxy for the likelihood that a DDI-induced ADR will occur. We further examined whether we could categorize specific drug classes according to rate of ADR occurrence. Table 2 shows the percentage of drugs for a given ATC code and their rate of occurrence in clusters C1–C4. The relative abundance of a particular drug class in clusters C1 and C2 indicates that these drugs are preferentially associated with DDI-induced ADRs. In particular, we found frequently occurring ADRs among drugs targeting the cardiovascular (ATC code C) and musculo-skeletal (ATC code M) systems. Conversely, antineoplastic and immune-modulating agents (ATC code L) were preferentially associated with non DDI-induced ADRs.

**Table 2:** Drugs grouped by Anatomical Therapeutic Chemical (ATC) classification code and their relative distribution in clusters C1–C4 based on the similarity of their predicted adverse drug reactions induced by synergistic drug-drug interactions (ADRs)

ATC code	Classification	All drugs		C1 %	C2 %	C3 %	C4 %	ADR
		N	%					
A	Alimentary tract and metabolism	64	10	12	14	10	8	~
B	Blood and blood forming organs	12	2	2	1	1	4	~
C	Cardiovascular system	88	14	22	24	11	8	+ +
D	Dermatologicals	19	3	2	0	4	5	–
G	Genito/urinary system and sex hormones	28	5	0	4	6	7	–
H	Systemic hormonal preparations	9	1	0	1	<1	4	–
J	Anti-infectives for systemic use	34	6	6	7	5	5	~
L	Antineoplastic and immune-modulating agents	61	10	1	1	11	20	– –
M	Musculo-skeletal system	45	7	16	7	4	5	+ +
N	Nervous system	167	27	29	28	31	21	~
P	Anti-parasitic products, insecticides, and repellents	10	2	0	0	2	3	–
R	Respiratory system	49	8	6	11	8	7	~
S	Sensory organs	11	2	2	1	2	2	~
V	Various	16	3	3	2	3	2	~

Only drugs that have an ATC classification are included. The last column indicates the propensity of ADRs to be preferentially associated with compounds in clusters C1 and C2 (+ +), have no preferential association with drugs in any cluster (~), or be preferentially associated with compounds in clusters C3 and C4 (– or – –), respectively. Each column sums to 100% and the values indicate the distribution of drugs among the ATC classification for each cluster. The number of DDI-induced ADRs for each drug was highly variable, indicating no systematic variation of DDI-induced ADRs on a per drug basis among the ATC drug classes

#### Specific examples of drug and drug-class combinations

**Fig. 10:**
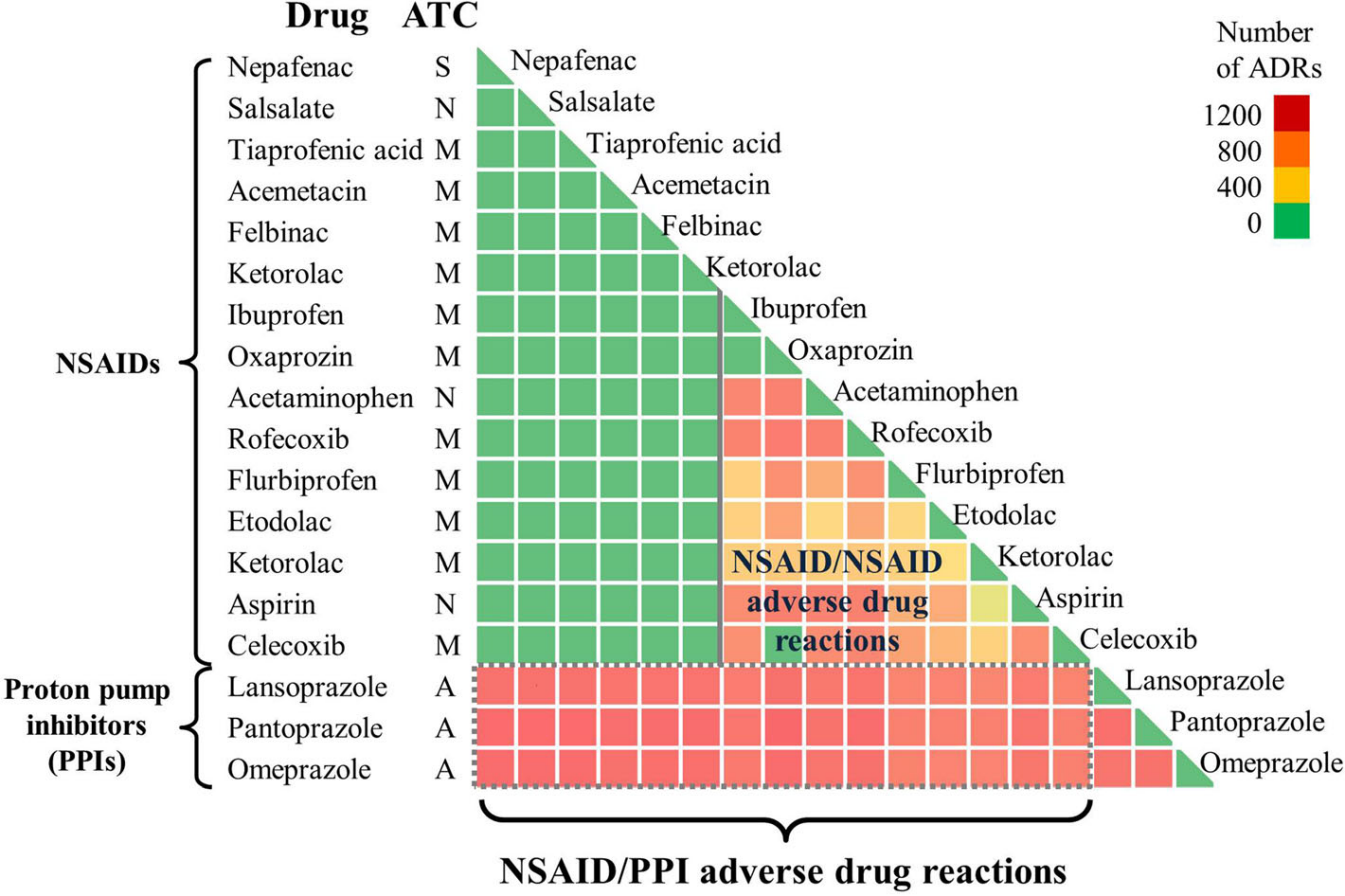
Class effect between proton pump inhibitors and non-steroidal anti-inflammatory drugs. All non-steroidal anti-inflammatory drugs exhibited DDI-induced ADRs with the proton pump inhibitors lansoprazole, pantoprazole, and omeprazole. ATC, anatomical therapeutic chemical classification; ADR, adverse drug reaction; NSAIDs, non-steroidal anti-inflammatory drugs; PPIs, proton pump inhibitors

Our analysis captured the DDI-induced ADRs known to occur between the anti-fungal drug ketoconazole and proton pump inhibitors (PPIs), such as omeprazole (Prilosec) [20]. PPIs alter gastric pH and thus reduce the anti-fungal activity of ketoconazole [20]. In our analysis, the PPIs pantoprazole, omeprazole, and lansoprazole ranked 1st, 2nd, and 10th, respectively, among drugs with the most DDI-induced ADRs associated with ketoconazole. This indicates that PPIs have the potential to induce a plethora of DDI-induced ADRs. Further analysis revealed a general class effect between these PPIs and non-steroidal anti-inflammatory drugs (NSAIDs). Figure 10 shows an increased number of DDI-induced ADRs associated with combining drugs from the two classes.

**Fig. 11:**
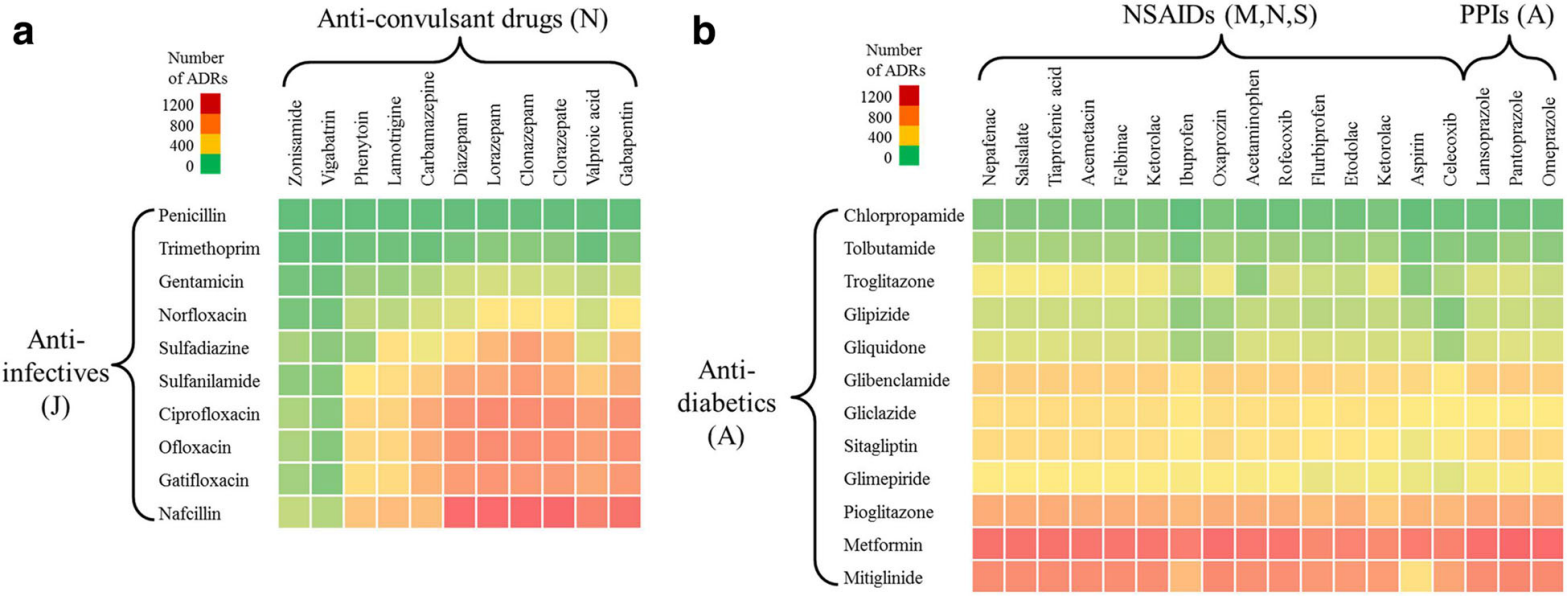
DDI-induced adverse drug reactions associated with specific drug classes. **a** Effects of jointly administering anti-infective (ATC code J) and anti-convulsant (ATC code N) drugs. **b** Effects of jointly administering anti-diabetics (ATC code A) and non-steroidal anti-inflammatory drugs (NSIADs, ATC codes M, N, and S) or proton pump inhibitors (ATC code A). ATC, anatomical therapeutic chemical classification; ADR, adverse drug reaction

We also predicted the likelihood of DDI-induced ADRs between anti-convulsant drugs (ATC code N) and anti-infective agents (ATC code J). Figure 11a shows that the anti-convulsant drugs gabapentin, valproic acid, lorazepam, diazepam, and carbamazepine were associated with relatively high numbers of DDI-induced ADRs when taken together with commonly used anti-infective agents, such as ciprofloxacin and ofloxacin. Conversely, other anti-convulsant drugs, such as zonisamide and vigabatrin, showed a low rate of ADR occurrence with anti-infective drugs. Widely prescribed anti-infectives, including ciprofloxacin, ofloxacin, gatifloxacin, and norfloxacin, belong to the class of fluoroquinolone anti-bacterial agents [21]. Among these, norfloxacin had a lower adverse DDI score when combined with an anti-convulsant than did ciprofloxacin or ofloxacin. Nafcillin, a penicillin-based anti-infective drug, had the highest number of ADRs when combined with an anticonvulsant. Our analyses suggest that when there is an option to choose an anti-infective for patients on treatment with anti-convulsant drugs, norfloxacin is preferable to ciprofloxacin. Similarly, Fig. 11b shows that the number of DDI-induced ADRs associated with anti-diabetic drugs (ATC code A) and common NSAIDs or PPIs varied widely. Anti-diabetic drugs, such as pioglitazone, metformin, and mitiglinide, were associated with a large number of DDI-induced ADRs, whereas alternatives, such as chlorpropamide, tolbutamide, and troglitazone, had a lower propensity to cause such ADRs. Thus, our analyses and tools can suggest alternative drugs that, when taken in combination with a required drug, may lower the likelihood of DDI-induced ADRs.

#### Spontaneous abortion, vestibular disorder, heat stroke, and rheumatic heart disease

**Fig. 12:**
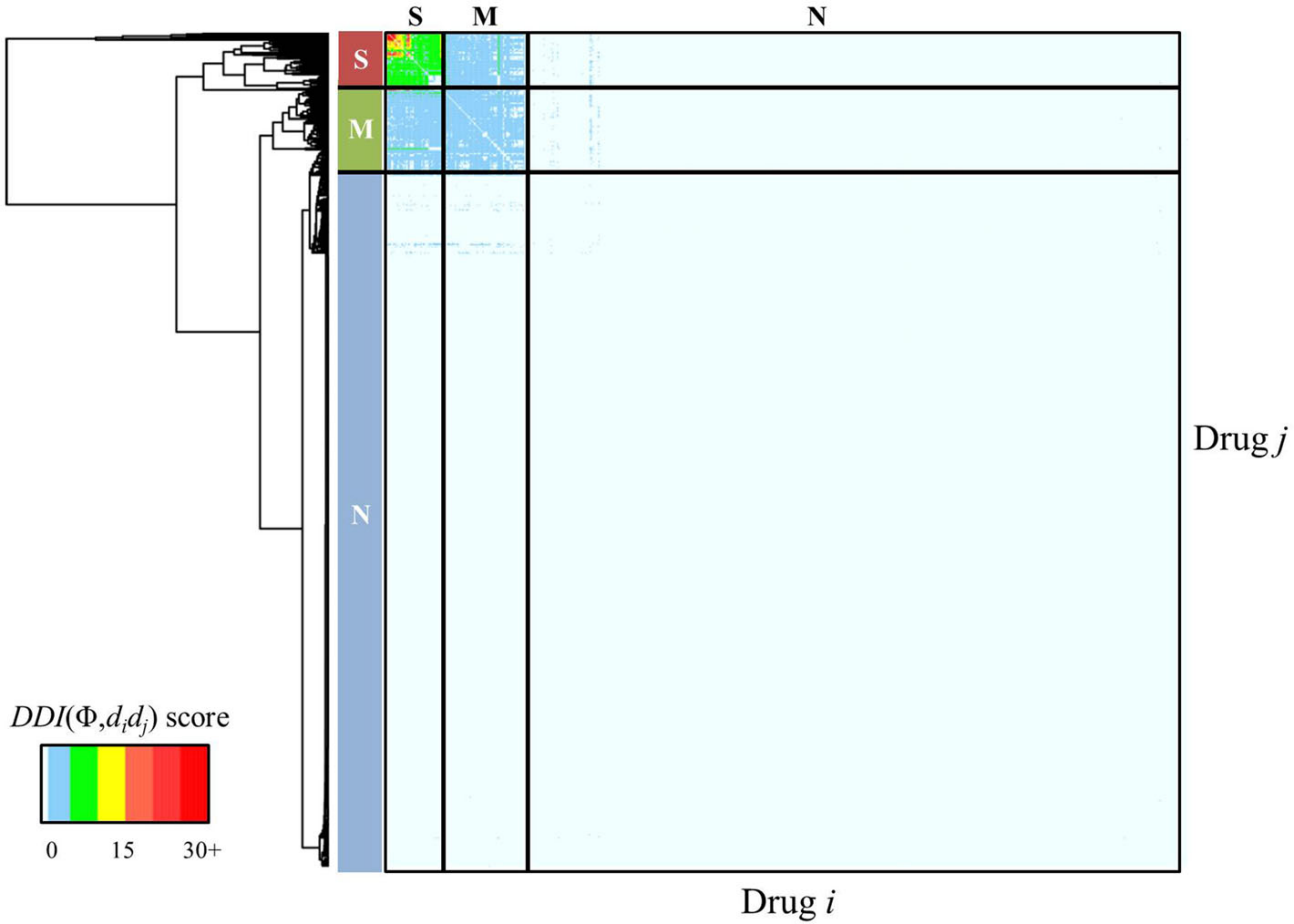
Two-way clustering of spontaneous abortion DDI scores. We included all drugs on the market for which we could make predictions. Each element in the matrix represents the *DDI*(Φ,*d*_*i*_ *d*_*j*_) score as calculated from equation (3) for causing spontaneous abortion (Unified Medical Language System C0000786) for each drug pair. The compounds roughly formed three drug-pair *categories*: *N*, those that do not cause spontaneous abortion; *M*, those with moderately high *DDI*(Φ,*d*_*i*_ *d*_*j*_) scores; and *S*, those associated with the highest scores

We examined in detail the drug combinations that were associated with the ADRs listed in Table 1. In this analysis, we used the calculated score for the particular ADR to cluster drug combinations. Figure 12 shows the compounds clustered by the score for inducing spontaneous abortion as predicted from all drug combinations, where each element in the matrix represents the *DDI*(Φ,*d*_*i*_ *d*_*j*_) score as calculated from equation (3). The drugs could roughly be grouped into three main categories denoted as *N*, *M*, and *S*, which represent compounds with little propensity to cause spontaneous abortion, those with moderately higher scores, and those with the highest scores, respectively. For spontaneous abortion, 97.1% of all drug combinations were associated with *category N*, 2.3% with *category M*, and 0.6% with *category S*. This indicates that most drug combinations were not strongly associated with high *DDI*(Φ,*d*_*i*_ *d*_*j*_) scores for spontaneous abortion.

Table 3 entries reflect the relative enrichment of an ATC class of compounds within a specific severity level of a given ADR. For example, of all the compounds classified as strongly (“*S*”) associated with inducing the ADR “Spontaneous abortion,” 63% belong to ATC *M* “Nervous system” compounds. This information captures what drug classes are most frequently associated with a DDI-induced ADR. Furthermore, this table also shows entries in bold, providing a visual means to gauge the enrichment of the severity level (moderate *M*, strong *S*) compared to the absence of a DDI-induced ADR (none *N*) for a given ATC compound class.

**Table 3:** Predicted distribution of different drug classes associated with four adverse drug reactions (ADRs)

ATC Classification		Spontaneous abortion			Vestibular disorder			Heat stroke			Rheumatic heart disease		
		*N*	*M*	*S*	*N*	*M*	*S*	*N*	*M*	*S*	*N*	*M*	*S*
		(%)			(%)			(%)			(%)		
A	Alimentary tract and metabolism	10	13	13	12	2	7	10	14	9	11	9	5
B	Blood and blood forming organs	2	0	2	2	0	0	3	0	0	2	0	0
C	Cardiovascular system	17	1	11	9	**35**	**39**	18	10	3	11	**45**	**24**
D	Dermatologicals	3	1	2	4	2	0	4	1	1	3	2	0
G	Genito/urinary system and sex hormones	4	**11**	0	5	3	5	6	4	1	5	0	0
H	Systemic hormonal preparations	2	0	0	2	2	0	2	0	0	2	2	0
J	Anti-infectives for systemic use	7	4	0	6	9	0	6	8	4	6	2	0
L	Antineoplastic and immune-modulating agents	12	0	2	12	5	0	15	0	0	10	9	5
M	Musculo-skeletal system	8	6	4	6	**14**	8	8	7	6	7	6	7
N	Nervous system	20	**47**	**63**	27	15	**40**	14	**31**	**69**	25	19	**60**
P	Anti-parasitic products, insecticides, and repellents	2	2	0	2	0	0	2	2	0	2	0	0
R	Respiratory system	7	**15**	4	9	8	0	6	**18**	6	9	0	0
S	Sensory organs	2	0	0	2	3	2	2	4	0	2	0	0
V	Various	3	0	0	3	3	0	4	0	1	2	6	0

We classified the drugs by two-way clustering of their DDI-induced ADR scores calculated from equation (3) into three categories: no ADR induction (*category N*), moderate ADR induction (*category M*), and strong ADR induction (*category S*). Fractions in *categories M* and *S* that are twice as large as the fraction in *category N* are indicated in bold. The columns sum to 100%, and the values characterize the distribution of drugs among the Anatomical Therapeutic Chemical (ATC) classifications for each cluster

The underlying numbers used to construct Tables 2 and 3 are provided in Additional file 1: Tables S1 and S2.

#### Bladder cancer as a DDI-induced ADR

Bladder cancer is one of the commonly occurring cancers among men in developed countries [22]. Two high-scoring drug combinations for inducing bladder cancer were pioglitazone-simvastatin and pioglitazone-metformin, with normalized DDI scores of 73.3 and 58.1, respectively. A review of the literature showed that pioglitazone, an anti-diabetic drug belonging to the class of thiazolidinediones, is associated with an increased risk of bladder cancer [23, 24]. Simvastatin is an extensively prescribed cholesterol-lowering drug of the statin family of 3-hydroxy-3-methylglutaryl-coenzyme A reductase inhibitors. Vinogradova et al. reported an increased risk of bladder cancer in patients taking statins for more than 4 years [14], whereas others have reported no association between statin use and increased risk of any cancer, including that of the bladder [25]. We found no studies on the potential DDI associated with thiazolidinediones and statins. Our analysis showed that the combined use of pioglitazone and simvastatin has the potential to induce or enhance bladder cancer through DDIs. Because these are drugs in common use, an analysis of this drug combination for risk of bladder cancer complications would be useful.

### Limitations

We based our analysis on the assumption that the drug-protein interaction profile carries important pharmacological and toxicological information that can be related to ADRs. This assumption might not hold for adverse effects that cannot primarily be traced back to events mediated by or through drug-protein interactions (e.g., those due to drugs that have a high intrinsic affinity for non-proteins, such as DNA, or have a high affinity for cellular fractions limited in the diversity of proteins, such as specific membrane- or fat-fractions). At the same time, drugs that lack such protein interactions will have few or possibly no entries in the STITCH database. Consequently, we were unable to instantiate such models.

The developed models provided categorical predictions of ADRs due to synergistic DDIs without considering dose or regimens as a factor. All drugs are toxic at sufficiently high doses; likewise, if drugs are taken in sufficiently small doses or for very short durations, their combination may not induce an ADR. Thus, the predictive ADR models provide information on the potential of particular drug combinations to induce an ADR rather than the actual event itself. Similarly, our models do not account for the susceptibility, sensitivity, or general physical health of individual patients, even though they may play an important role in individual cases.

We trained the models to predict ADRs for pairwise drug combinations. However, patients may actually be taking more than just two drugs at any time—a situation that could affect the outcome. Although the computational framework allows for including any number of combinations in the drug-protein profiles, we do not have sufficient information to validate these predictions. Accordingly, we limited the scope of the current work to include only pairwise combinations.

## Conclusions

ADRs cause considerable morbidity and mortality despite the extensive pre-clinical and clinical studies that precede drug approval and marketing. ADRs due to combinations of two or more drugs are especially insidious because prospective testing and evaluation of drug safety for all possible combinations is impossible; consequently, instances and warnings of DDI-induced ADRs are usually generated after drug approval. This means that significant risks of experiencing these ADRs are actually borne by patient populations most likely to take multiple drugs—for example, the elderly and other vulnerable populations affected by comorbidities.

A robust *in silico* method that predicts ADRs would provide an easily accessible tool to evaluate and avoid these effects. The method we developed in this work is robust with respect to false positives and false negatives in the clinical data used to build the models. The final models deployed had an overall mean accuracy of 89%, sensitivity of 63%, and specificity of 90%.

We made categorical predictions for pairwise combinations of nearly 800 drugs presently on the market. The predictions indicated that 90% of the combinations are unlikely to cause DDI-induced ADRs. Conversely, the results suggest that of the potential ADRs associated with 10% of the combinations, only a small fraction is clinically recognized at present. Our analyses using the rate of occurrence as well as the DDI scores associated with model predictions revealed drug classes highly likely to be involved in DDI-induced ADRs, such as those targeting the cardiovascular or musculo-skeletal systems. The models also captured class effects between different categories of drugs, such as NSAIDs and PPIs, as well as between different types of therapeutic targets, such as anti-infective and anti-convulsants. Such information could be used to select combinations that avoid potential ADRs, or suggest alternative therapeutics that minimize the number of DDI-induced ADRs. As an example, we also examined DDI-induce bladder carcinoma predictions and identified drug combinations supported by the literature but not yet reported in the clinic. These results highlight the potential prospective use of our models in pharmacovigilance. Furthermore, the Web-accessible and searchable database developed here provides a means to quickly examine and download the results of our predictions for a particular drug, drug combination, or potential adverse health effect.

## Additional file

Additional file 1:
**Figure S1.** and **Tables S1.** and **S2.** (DOCX 7762 kb)

## Abbreviations

ADR: Adverse drug reaction
ATC: Anatomical therapeutic chemical classification
AUC: Area under the curve
AVOID-DB: AdVerse effects Of Interacting Drugs Database
DDI: Drug-drug interactions
DPIR: Drug-protein interaction profile-based repurposing
GUI: Graphical user interface
NSAID: Non-steroidal anti-inflammatory drugs
PPI: Proton pump inhibitor
UMLS: Unified medical language system
