# Supplementary material for: Data-driven prediction of adverse drug reactions induced by drug-drug interactions
Source: BMC Pharmacol Toxicol. 2017 Jun 8;18:44. doi: 10.1186/s40360-017-0153-6 (PMC5465578; doi:10.1186/s40360-017-0153-6)
Supplement: Additional file 1: — Figure S1. and Tables S1. and S2. (DOCX 7762 kb) [file 40360_2017_153_MOESM1_ESM.docx]

Figure S1. Clustering of all drug combinations based on DDI scores for the four exemplar ADRs.

| **ATC code** | **Classification** | **All** | | **C1 (n)** | **C2 (n)** | **C3 (n)** | **C4 (n)** |
| --- | --- | --- | --- | --- | --- | --- | --- |
|  |  | **N** | **%** |  |  |  |  |
| A | Alimentary tract and metabolism | 64 | 10 | 14 | 15 | 22 | 13 |
| B | Blood and blood forming organs | 12 | 2 | 2 | 1 | 3 | 6 |
| C | Cardiovascular system | 88 | 14 | 25 | 25 | 25 | 13 |
| D | Dermatologicals | 19 | 3 | 2 | 0 | 9 | 8 |
| G | Genito/urinary system and sex hormones | 28 | 5 | 0 | 4 | 13 | 11 |
| H | Systemic hormonal preparations | 9 | 1 | 0 | 1 | 1 | 7 |
| J | Anti-infectives for systemic use | 34 | 6 | 7 | 7 | 12 | 8 |
| L | Antineoplastic and immune-modulating agents | 61 | 10 | 1 | 1 | 26 | 33 |
| M | Musculo-skeletal system | 45 | 7 | 19 | 7 | 10 | 9 |
| N | Nervous system | 167 | 27 | 34 | 29 | 70 | 34 |
| P | Anti-parasitic products, insecticides, and repellents | 10 | 2 | 0 | 0 | 5 | 5 |
| R | Respiratory system | 49 | 8 | 7 | 12 | 19 | 11 |
| S | Sensory organs | 11 | 2 | 2 | 1 | 5 | 3 |
| V | Various | 16 | 3 | 3 | 2 | 7 | 4 |

Table S1. Drugs grouped by the Anatomical Therapeutic Chemical (ATC) classification code and their absolute distribution in clusters C1–C4 based on the similarity of their predicted adverse drug reactions induced by synergistic drug-drug interactions (ADRs). The corresponding relative numbers are given in Table 2.

| **ATC classification** | | **Spontaneous abortion** | | | **Vestibular disorder** | | | **Heat stroke** | | | **Rheumatic heart disease** | | |
| --- | --- | --- | --- | --- | --- | --- | --- | --- | --- | --- | --- | --- | --- |
|  |  | **N** | **M** | **S** | **N** | **M** | **S** | **N** | **M** | **S** | **N** | **M** | **S** |
| **A** | Alimentary tract and metabolism | 48 | 9 | 7 | 59 | 1 | 4 | 41 | 13 | 10 | 58 | 4 | 2 |
| **B** | Blood and blood forming organs | 11 | 0 | 1 | 12 | 0 | 0 | 12 | 0 | 0 | 12 | 0 | 0 |
| **C** | Cardiovascular system | 81 | 1 | 6 | 42 | 23 | 23 | 74 | 10 | 4 | 57 | 21 | 10 |
| **D** | Dermatologicals | 17 | 1 | 1 | 18 | 1 | 0 | 17 | 1 | 1 | 18 | 1 | 0 |
| **G** | Genito/urinary system and sex hormones | 20 | 8 | 0 | 23 | 2 | 3 | 23 | 4 | 1 | 28 | 0 | 0 |
| **H** | Systemic hormonal preparations | 9 | 0 | 0 | 8 | 1 | 0 | 9 | 0 | 0 | 8 | 1 | 0 |
| **J** | Anti-infectives for systemic use | 32 | 3 | 0 | 29 | 6 | 0 | 22 | 8 | 5 | 34 | 1 | 0 |
| **L** | Antineoplastic and immune-modulating agents | 60 | 0 | 1 | 58 | 3 | 0 | 61 | 0 | 0 | 55 | 4 | 2 |
| **M** | Musculo-skeletal system | 39 | 4 | 2 | 31 | 9 | 5 | 31 | 7 | 7 | 39 | 3 | 3 |
| **N** | Nervous system | 99 | 34 | 34 | 133 | 10 | 24 | 57 | 30 | 80 | 133 | 9 | 25 |
| **P** | Anti-parasitic products, insecticides, and repellents | 9 | 1 | 0 | 10 | 0 | 0 | 8 | 2 | 0 | 10 | 0 | 0 |
| **R** | Respiratory system | 36 | 11 | 2 | 44 | 5 | 0 | 25 | 17 | 7 | 49 | 0 | 0 |
| **S** | Sensory organs | 11 | 0 | 0 | 8 | 2 | 1 | 7 | 4 | 0 | 11 | 0 | 0 |
| **V** | Various | 15 | 0 | 0 | 13 | 2 | 0 | 14 | 0 | 1 | 12 | 3 | 0 |

**Table S2**. Predicted absolute distribution of different drug classes associated with four adverse drug reactions (ADRs). We classified the drugs by two-way clustering of their DDI-induced ADR scores calculated from equation (3) into three categories: no ADR induction (*category N*), moderate ADR induction (*category M*), and strong ADR induction (*category S*). The relative distribution is shown in Table 3.


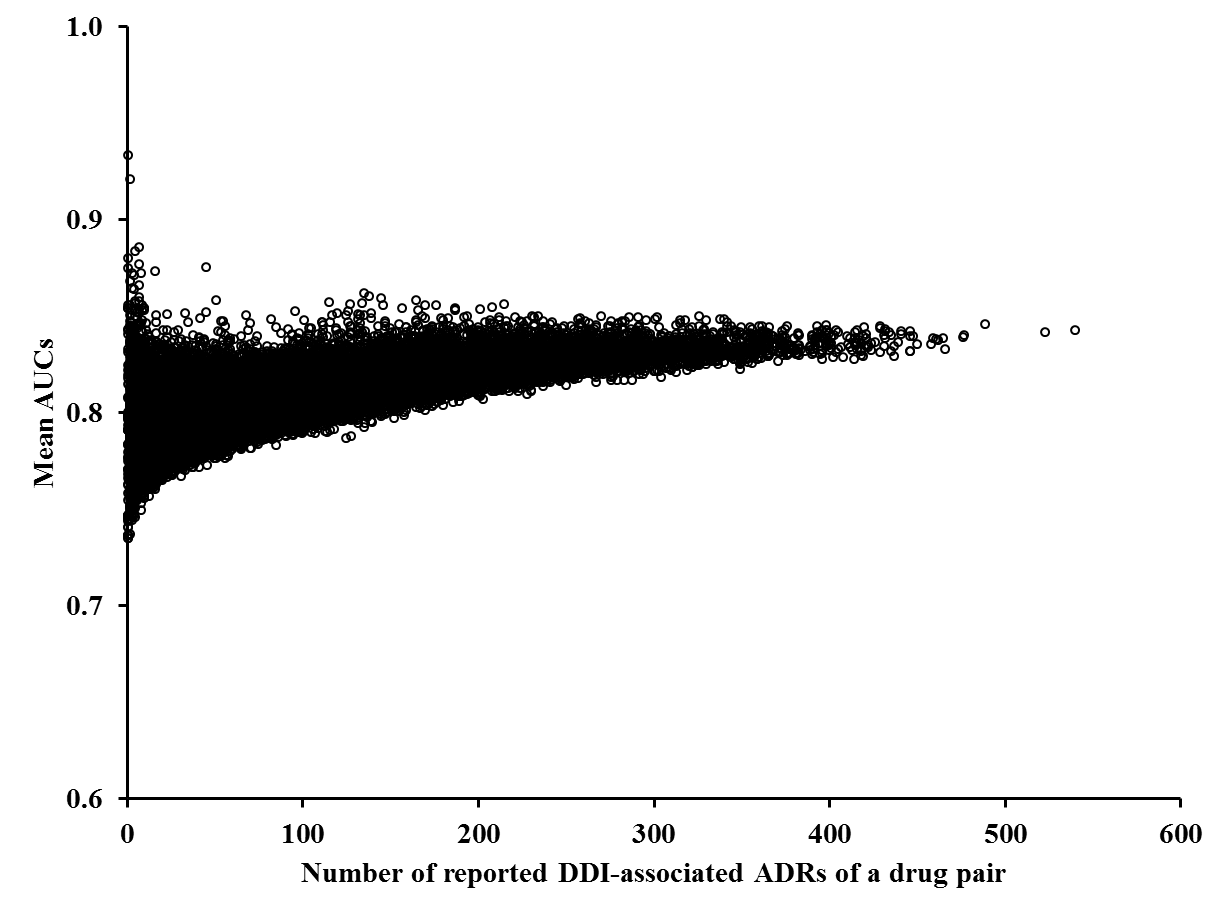


Figure S2. Performance of DDI-associated ADR prediction models as a function of the number of reported ADRs for a drug pair. The abscissa shows the number of ADRs associated with each drug pair in the TWOSIDES database. The ordinate shows the mean AUC values of the ADR models for each drug pair. The lack of an apparent up- or down-ward trend indicates that the reported frequency of ADRs for a drug pair does not affect model performance.
